# Supplementary material for: Lipid-mediated PX-BAR domain recruitment couples local membrane constriction to endocytic vesicle fission
Source: Nat Commun. 2017 Jun 19;8:15873. doi: 10.1038/ncomms15873 (PMC5481832; doi:10.1038/ncomms15873)
Supplement: Supplementary Information [file ncomms15873-s1.pdf]

Type of file: pdf
Title of file for HTML: Supplementary Information
Description: Supplementary Figures, Supplementary Tables, and Supplementary References

Type of file: mp4
Title of file for HTML: Supplementary Movie 1
Description: Neck constriction and Snx9 recruitment simulation. SNX9 tip-to-tip interactions induce oligomer formation.

Type of file: pdf
Title of file for HTML: Peer Review File
Description:

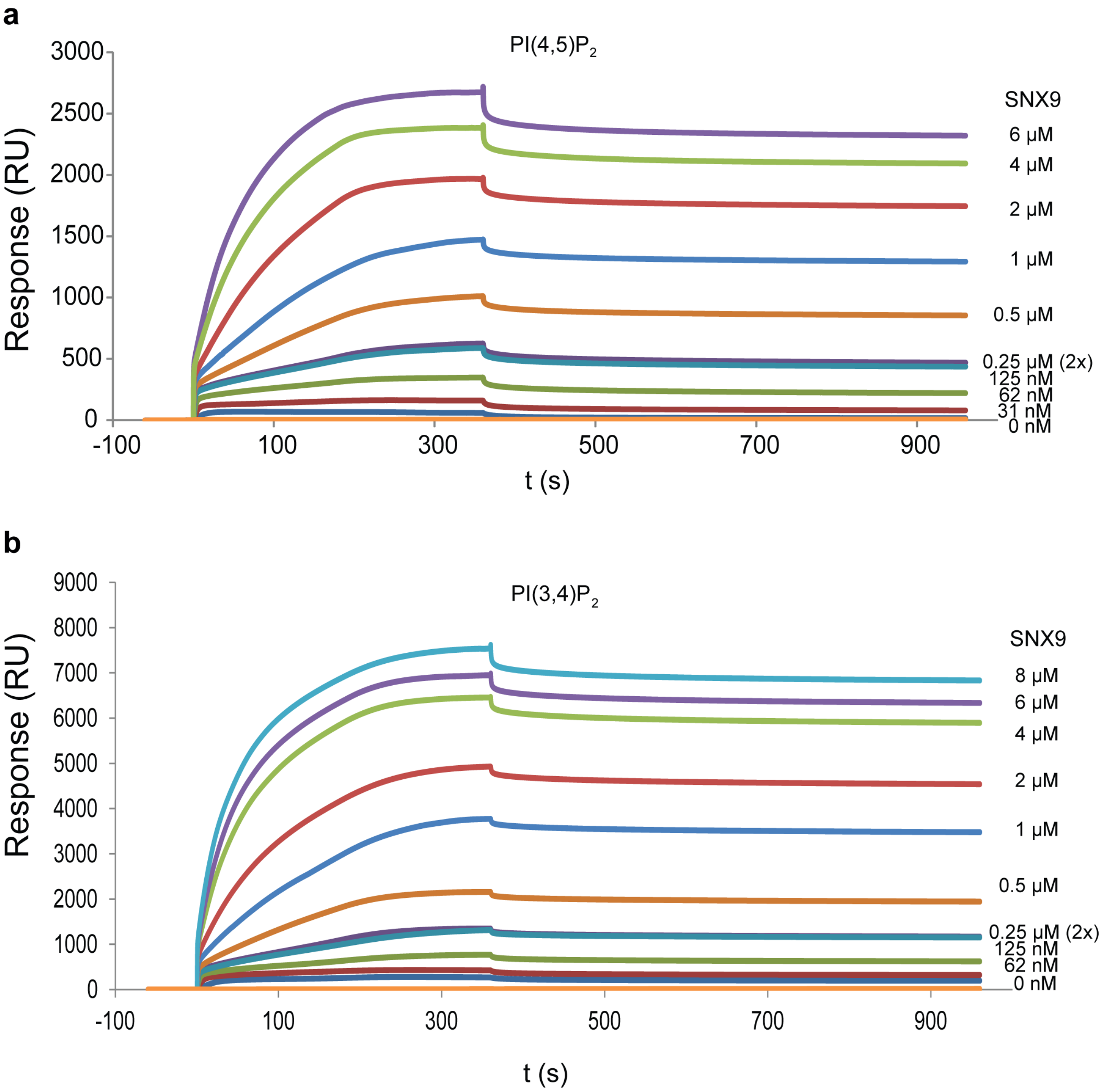

Supplementary Figure 1 | Binding of SNX9 (PX-BAR) to PI(4,5)P$_2$ or PI(4,5)P$_2$. Binding of SNX9 (PX-BAR) to (**a**) PI(4,5)P$_2$ or (**b**) PI(3,4)P$_2$ was recorded with an L1 biosensor chip using the Biacore^{TM} T200 system. L1 biosensor chips contained immobilized liposomes composed of PC:PE (70%:30%, w/w) as a reference channel or PC:PE:PIP$_2$ (68%:30%:2%, w/w) at a level of 6,000-10,000 RU. Sensorgrams show concentration-dependent binding of SNX9 (PX-BAR).

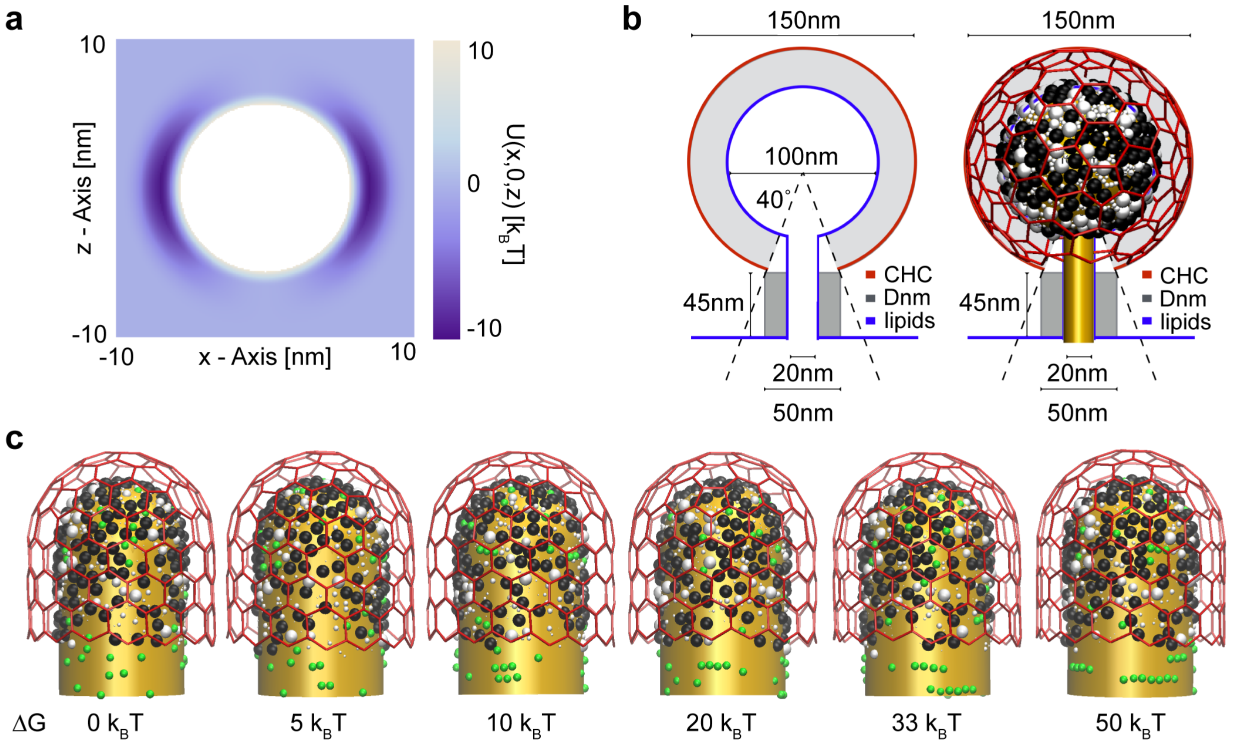

Supplementary Figure 2 | SNX9 oligomerization affinity leads to SNX9 ring formation.
(**a**) To model the oligomerization of SNX9 molecules, an interaction potential for SNX9 was derived that is repulsive in its core (white) and attractive in the x-y plane (dark blue). The directionality of the potential models the fact that SNX9 can only bind to membranes with a suitable curvature and will therefore be roughly aligned in the same manner (e.g. tube with curvature along x-y leads to alignment along x-y). The most important potential parameter is the well depth that determines the SNX9-SNX9 binding affinity. (**b**) Geometry of the constricted state. (**c**) Simulations were conducted using different interaction energy well depths d between 0 k_BT and 50 k_BT (1kbT ~ 0.6 kcal/mol). Based on Simulations (Fig 2b), 42 SNX9 copies present after $PI(3,4)P_2$ recruitment are simulated, which are located at the coat or at the neck. We find that values of ~10 k_BT, corresponding to the formation of two salt bridges[1], to be sufficient to form linear SNX9 assemblies, that at narrow necks will lead to rings (Fig. 4e). Deeper potential wells lead to the formation of more stable SNX9 chains.

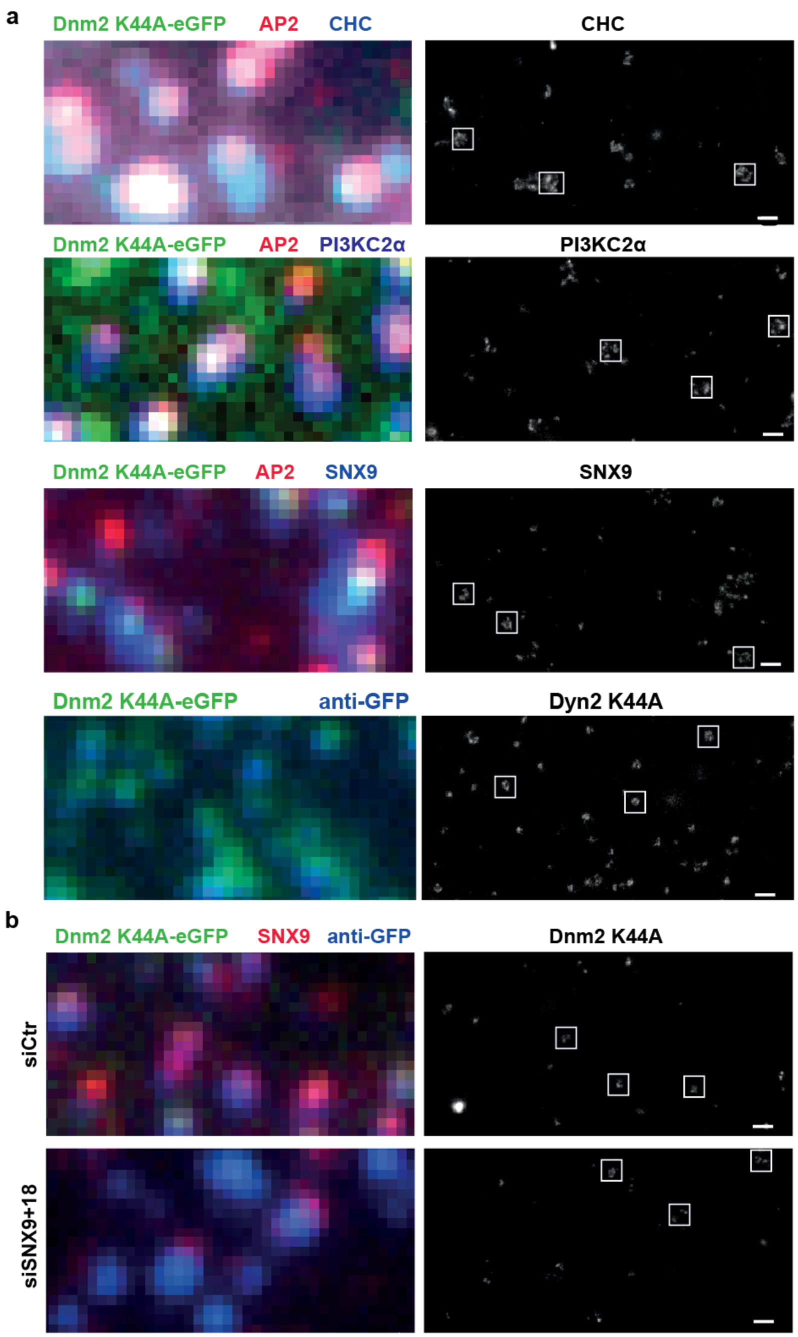

Supplementary Figure 3 | dSTORM imaging of endogenous proteins at late-stage CCP in Cos7 cells. (**a**,**b**) Widefield (left) and dSTORM images (right) of Cos7 cells expressing Dnm2-K44A GFP. (**a**) Endogenous clathrin heavy chain (CHC), PI3KC2α, SNX9 and overexpressed Dnm2-K44A-GFP (anti-GFP) were stained by indirect immunofluorescence using CF647-conjugated secondary antibodies. AP-2 was stained by anti-α-adaptin (AP6, Abcam ab2730) using CF568 conjugated secondary antibodies. (**b**) Overexpressed Dnm2-K44A-eGFP in control and SNX9/18 depleted cells was stained by indirect immunofluorescence using CF647-conjugated secondary antibodies. Endogenous SNX9 was stained by anti-α-SNX9 and CF568 conjugated secondary antibodies. Scale bars 200nm. White boxes mark examples of regions that where used for center-alignment of the structures.

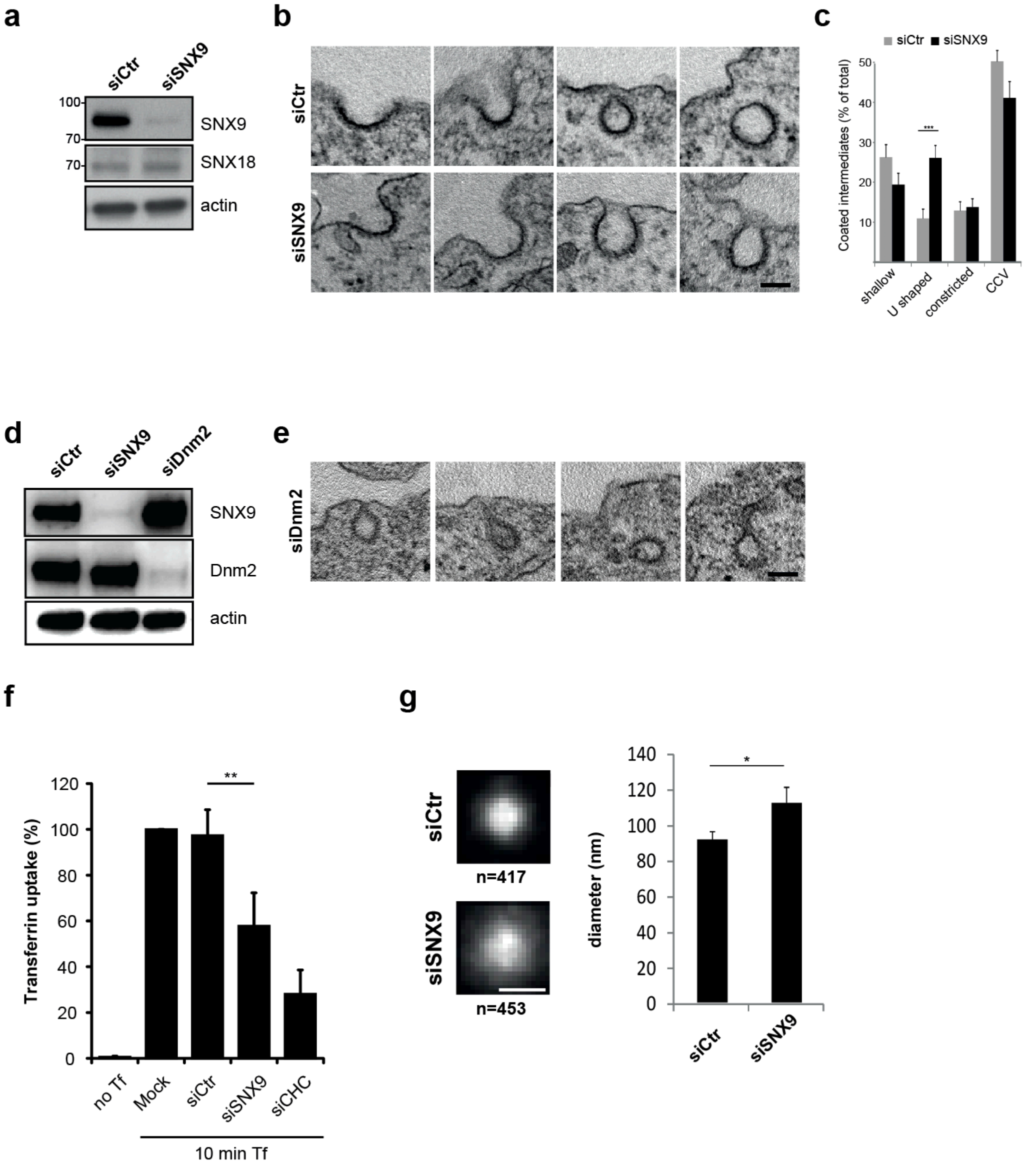

Supplementary Figure 4 | Knockdown of SNX9 results in late maturation defects and reduced CME in HeLa cells. (**a**) Efficient siRNA-mediated depletion of SNX9 in HeLa cells. (**b, c**) Ultrastructural analysis of CCPs in control or SNX9-depleted cells. Morphological groups were shallow, non-constricted U-shaped, constricted Ω-shaped pits, or structures containing complete clathrin coats. (**b**) Representative images from controls (top) or a SNX9-depleted cells illustrating accumulation of U-shaped pits (bottom). Scale bar, 100 nm. (**c**) Bar diagram detailing the relative abundance of different clathrin-coated structures in (b) control vs. SNX9-depleted HeLa (mean ± s.e.m.; n = 30 (siCtr) or n = 33 (SNX9 siRNA) cell perimeters, two-sided Student's t-test,*** $p<0.001$). (**d**) Efficient siRNA-mediated depletion of SNX9 or Dnm2 in HeLa cells. (**e**) Representative images from Dnm2-depleted cells illustrating accumulation of constricted pits with short and long necks (**f**) Impaired CME of transferrin in HeLa cells depleted of SNX9 was measured by FACS after internalization of Tf-AF647 for 10 min at 37°C (mean ± s.e.m.; n=5 experiments, two-sided Student's t-test,** $p<0.01$) (**g**) dSTORM center-aligned average images and diameter of GFP-Dnm2 K44A in HeLa cells depleted of SNX9 (mean ± SD, n=5 cells, 417-453 CCPs, two-sided Student's t-test,* $p<0.05$). Scale bars 100nm.

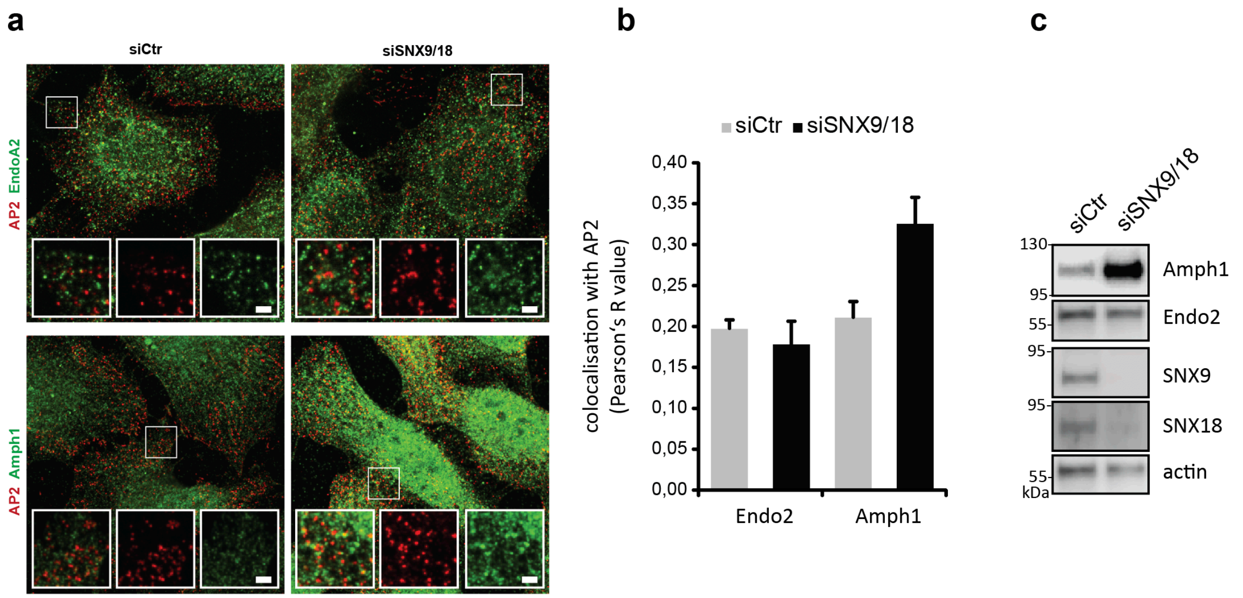

Supplementary Figure 5 | Amphiphysin 1 is upregulated in SNX9/18 depleted cells

(**a**) Representative confocal images illustrating the localization of AP-2 and endophilin A2 or amphiphysin 1 in methanol fixed control cells or cells depleted of SNX9/18. Scale bar, 1μm

(**b**) Colocalization analysis for AP-2 and endophilin A2 or amphiphysin 1 proteins using Pearson's R value (mean (± SEM) from 14-16 field from two independent experiments, Coloc2 ImageJ, NIH).

(**c**) Immunoblot analysis of control cells (siCtr) or cells depleted of SNX9/18 using antibodies against the indicated proteins. Endo2, endophilin A2, Amph1, amphiphysin 1.

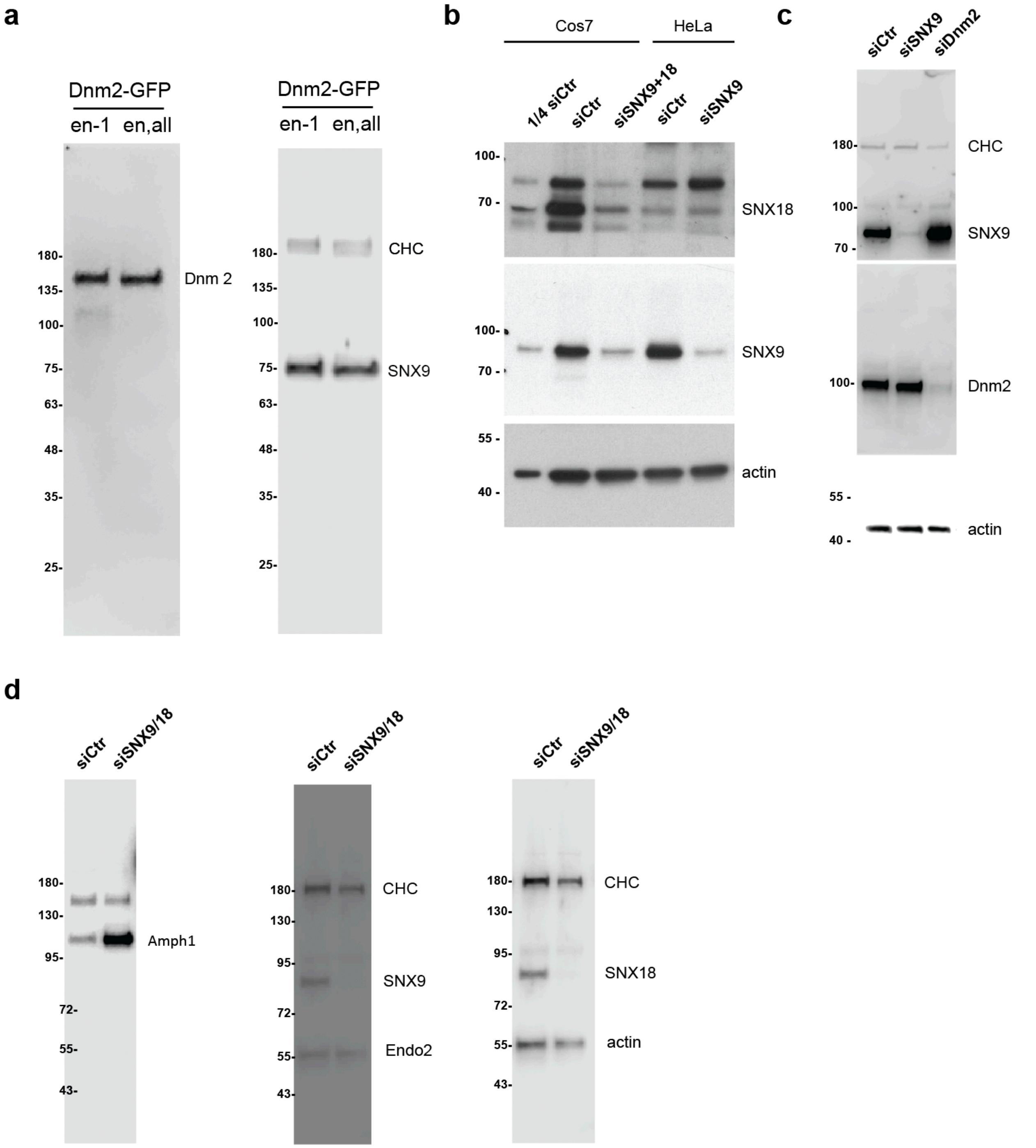

Supplementary Figure 6 | Uncropped Images of Western Blots
(**a**) Uncropped Immunoblots from Fig 1g
(**b**) Uncropped Immunoblots from Fig 6a and Ext. Fig 4a
(**c**) Uncropped Immunoblots from Ext. Fig 4d
(**d**) Uncropped Immunoblots from Ext. Fig 5c Endo2, endophilin A2, Amph1, amphiphysin 1.

Supplementary Table 1 | Reference parameter set for the large CCP RDME

Parameters for a protein recruitment simulation of a CCP corresponding to an endocytic vesicle of 100 nm membrane diameter and 150 nm diameter of the clathrin cage consisting of 180 clathrin triskelia. Except for experimentally determined proteins (Fig 1h) protein copy numbers are derived from proteomic studies[2]. In the case of SNX9 peak recruitment was determined in genome-engineered SK-MEL-2 cells (see Figure 1h). We estimate that about half of the final number of SNX9 molecules is present prior to $PI(3,4)P_2$ synthesis by PI3KC2α. SNX9, AP-2, CALM and FCHo2 dimers are modeled explicitly, other PIP binders (ARH, PI3KC2α, Hip1, Hip1R, Dab2 and Epsin) are combined into the category ('Other PIP Binders') to simulate the lipid competition. The footprint size of members from this category is chosen such that the total occupied protein area is the same as in the proteomic study. (**a**) Experimentally-determined parameters used for the proteins simulated in the reaction-diffusion model, references are marked by superscripts and enlisted below. (**b**) Protein-specific parameters used in the reaction-diffusion simulation that were either derived from (a), or computed as described in SI methods. (**c**) Experimental and model parameters for the two phospholipids. (**d**) Global simulation parameters. (**e**) Kinase and phosphatase turnover rates. The phosphatase was not active in the reference parameter set, but only in some alternative parameter set considered in the parameter sampling (see Extended Data Table 3).

**a)**	**SNX9**	**AP-2**	**CALM**	**FCHo2**	**Other PIP Binders**
**copy number** [1]	22	180	180	10	198
**footprint [nm²]** [2]	34.25	84.5	8.46	83.6	9.8
**# lipid bind. sites**	2	4 (2s, 2w)[3]	1	2	1
**$K_{D,45}$ [µMol]**	1.1	7.6 (30.0)[3,4]	5.8[5]	7[6]	0.86[7]
**$K_{D,34}$ / $K_{D,45}$**	1.36	7.7[4]	4.54[8]	4[9]	4[8]

b)	SNX9	AP-2	CALM	FCHo2	Other PIP Binders
k_{diff} [s^{-1}]	6741.57	3370.79	13483.1	6741.57	13483.1
$conc_{cytosol}$ [$1/A_{Sim}$]	0.055	18.5	1.18	0.27	0.21
$K_{a,mem}$	0.1	5	5	0.1	5
$K_{a,lip45}$	9.0	0.2	10.5	3.6	71.2
$K_{a,lipweak45}$		0.0055			
$K_{a,lip34}$	7.7	0.06	2.3	1.8	17.8
$K_{a,lipweak34}$		0.0078			
$K_{a,clathrin}$	1.0	1.0	1.0	1.0	1.0

c)	D [µm²/s] (experiment)	k_{diff} [s^{-1}] (model)	init. $PI(3,4)P_2$ [μm^{-2}]	init. $PI(4,5)P_2$ [μm^{-2}]
**Phospholipids ($PI(3,4)P_2$, $PI(4,5)P_2$)**	3[10]	13483.1	0	13,750

d)	time [s]	dt [µs]	cellsize [nm^2]	cellradius [nm]	# cells	total sim. area A_{sim} [μm^2]	$k_{on,membrane}$ [s^{-1}]	$k_{on,lipid}$ [s^{-1}]	$k_{on,clathrin}$ [s^{-1}]
**Global**	60	10	222.5	9.25	547	0.12	100000	10000	10

e)	k_{kinase} [s^{-1}]	$k_{phosphatase}$ [s^{-1}]
**Clathrin N-terminal domain**	4	(0.26)

Supplementary Table 2 | Parameter sampling results. Simulation parameters for which quantitatively reliable measurements were not available were varied in order to test whether our conclusions were robust with respect to parameter changes. For visual clarity, the 162 simulation results are grouped to sets in which a single parameter is constant. Each row reports the averages and standard deviations over the variable parameters in the corresponding set of simulations. To ensure sufficient statistics, all 162 possible combinations of parameter values were simulated for 4 runs, resulting in 648 simulation runs total.

The first column reports the parameters considered for sampling and is kept constant in the corresponding row. CA - The affinity of all proteins to bind the N-terminal domain of clathrin. PI(4,5)P_2 affinity - The affinity of SNX9 to PI(4,5)P_2 in relation to PI(3,4)P_2. PI(4,5)P_2 depletion - the concentration of the lipid is depleted with a constant rate starting from t=0s (or not). [PI(4,5)P_2] - concentration of the lipid in the simulation. [PI(3,4)P_2 = x[PI(4,5)$_2$] - the target concentration of the lipid PI(3,4)P_2 in relation to the initial concentration of PI(4,5)P_2. Diffusion slowdown indicates the factor by which the lipid diffusion is reduced under the CCP as compared to free membrane. This parameter governs the final PI(3,4)P2 concentration. The parameter values of the reference parameter set are highlighted in green, i.e. Fig 2b corresponds to setting all five parameters to the green highlighted value.

The parameters were assessed based on the properties listed in the first row, columns 2-5. In all scenarios, the concentration of SNX9 at t=0s (before addition of PI(3,4)P_2) was below the putative threshold of around 40 copies required for ring formation (column 2). Upon PI(3,4)P_2 increase the concentration of SNX9 at t=40s increases (~2x). Higher PI(4,5)P_2 affinities and higher PI(3,4)P_2 concentrations have the most prominent effect on the final SNX9 concentration. While a certain SNX9 affinity to PI(4,5)P_2 is needed to ensure a baseline concentration of SNX9, the number of recruited SNX9 copies mainly depends on the amount of PI(3,4)P_2 produced (see Fig 1b & Extended Data Fig. 2). Column 4 reports the relative increase of SNX9 when dividing the SNX9 copy number after PI(3,4)P_2 production by the SNX9 copy number before the increase of PI(3,4)P_2. Low PI(4,5)P_2 concentrations also results in a high relative increase of SNX9, but are however starting from low initial SNX9 copy numbers and mostly do not reach sufficient total SNX9 copy numbers. Consequently, both the ability to bind to PI(4,5)P_2 and sufficient PI(3,4)P_2 levels are necessary to accumulate sufficient SNX9. Column 5 shows the relative change in copy numbers of the other proteins between t=0s and t=40s. Note that the other proteins remain unchanged upon PI(3,4)P_2 increase.

**Parameter**	**[SNX9] at t=0s**	**[SNX9] at t=40s**	**Increase SNX9**	**Increase other Proteins**
**CA = 0**	10.64 ±17.37	20.11 ±21.08	1.89	1.01 ±0.13
**CA = 1**	10.66 ±17.36	20.25 ±21.18	1.9	1.01 ±0.13
**CA = 100**	10.68 ±17.34	20.21 ±21.39	1.89	1.01 ±0.13
**PI(4,5)P_2 Affinity = 0**	0.04 ±0.01	6.56 ±5.6	164	1.01 ±0.13
**PI(4,5)P_2 Affinity = ½**	2.65 ±1.65	12.33 ±7.01	4.65	1.01 ±0.13
**PI(4,5)P_2 Affinity = 1**	29.28±19.43	41.68±23.67	1.42	1.01 ±0.13
**no PI(4,5)P_2 depletion**	10.64 ±17.32	22.21 ±24.1	2.09	1.11 ±0.11
**PI(4,5)P_2 depletion**	10.68±17.39	18.17 ±17.64	1.7	0.91 ±0.05
**[PI(4,5)P_2] = 6,750/µm²**	3.45 ±4.29	14.34 ±11.04	4.16	1.08 ±0.18
**[PI(4,5)P_2] = 13,500/µm²**	8.79 ±11.13	18.37 ±16.39	2.09	0.98 ±0.11
**[PI(4,5)P_2] = 27,000/µm²**	19.73 ±24.98	27.86±29.38	1.41	0.98 ±0.03
**Diffusion slowdown 100x**	8.95±14.5	11.59±14.93	1.29	0.97 ±0.11
**Diffusion slowdown 200x**	10.2±16.43	17.39±18.03	1.7	1.01 ±0.12
**[Diffusion slowdown 500x**	12.83±20.39	31.6 ±24.3	2.46	1.05 ±0.015

Supplementary Table 3 | Physical protein characteristics for the spatial model. Listed are all proteins that were used in the iPRD simulation model. As a reference value to calculate the effective particle radius in the model, either the known dimensions or the molecular weight were used - therefore either of the two values is reported in column 2, along with the corresponding reference in column 5. The radius used in the spatial model and the copy numbers are listed in columns 3 and 4, respectively. A conservative approach, i.e. lower copy numbers leading to less crowding is assumed. Therefore all copy numbers were taken from[2] and omitting the protein Numb. * indicates PIP binders summarized in the 'Other PIP binders' class in the RDME.

Protein	dimensions or weight	radius [nm]	copy number	Reference
SNX9	13.7 nm x 2.4 nm	3.3	initial 22 final 40	PDB 2RAI[11]
AP-2 complex open formation	10.3 nm x 8.2 nm	5.2	180	PDB 2xa7[3]
CALM	3.6 nm x 2.4 nm	1.7	180	PDB 1HFA[5]
FCHo2 dimer	20.9 nm x 4.0 nm	5.2	10	PDB 2V0O[12]
Epsin*	3.5 nm x 2.8 nm	1.7	20	PDB 1EDU[13]
ARH*	34 kDa	0.6	49	Uniprot
PI3KC2α*	190 kDa	3.6	33	Uniprot
NECAP1	30 kDa	0.6	38	Uniprot
Hip1*	116 kDa	2.2	32	Uniprot
Hip1R*	119 kDa	2.2	32	Uniprot
HRB	58 kDa	1.1	32	Uniprot
Dab2*	82 kDa	1.5	27	Uniprot
NECAP2	28 kDa	0.5	27	Uniprot

Supplementary Table 4 | Antibodies

antibody	species	clone	source	dilution IF	dilution WB
Amphyphysin	Rabbit	120002	Synaptic Systems	1:100	1:2000
B-actin	Mouse	ac-15	Sigma-Aldrich		1:10, 000
Clathrin heavy chain	Rabbit	ab21679	Abcam	1:200	1:500
Dynamin 1+2	Mouse	41	BDBiosciences	-	1:500
Endophilin 2	Rabbit	Stimpy	Pietro DeCamilli	1:200	
Endophilin 2	Rabbit	sc-25495	Santa Cruz		1:100
PI3K C2α	Rabbit	#58	Raised against aa 2-365 of human PI3K C2α	1:200	1:2,000
GFP	Mouse	3E6	Invitrogen A-11120	1:200	
GFP-CF680	Mouse		Biotium 20219	1:200	
SNX9	Rabbit		Protein Tech #15721-1-AP	1:100	1:500
SNX18	Rabbit	119426	GenTex #GTX119426	-	1:5,000

SUPPLEMENTARY REFERENCES

1 White, A. D. *et al.* Free energy of solvated salt bridges: a simulation and experimental study. *J Phys Chem B* **117**, 7254-7259, doi:10.1021/jp4024469 (2013).

2 Borner, G. H. *et al.* Multivariate proteomic profiling identifies novel accessory proteins of coated vesicles. *J Cell Biol* **197**, 141-160, doi:10.1083/jcb.201111049 (2012).

3 Jackson, L. P. *et al.* A large-scale conformational change couples membrane recruitment to cargo binding in the AP2 clathrin adaptor complex. *Cell* **141**, 1220-1229, doi:10.1016/j.cell.2010.05.006 (2010).

4 Honing, S. *et al.* Phosphatidylinositol-(4,5)-bisphosphate regulates sorting signal recognition by the clathrin-associated adaptor complex AP2. *Mol Cell* **18**, 519-531, doi:10.1016/j.molcel.2005.04.019 (2005).

5 Ford, M. G. *et al.* Simultaneous binding of PtdIns(4,5)P2 and clathrin by AP180 in the nucleation of clathrin lattices on membranes. *Science* **291**, 1051-1055, doi:10.1126/science.291.5506.1051 (2001).

6 Uezu, A. *et al.* Characterization of the EFC/F-BAR domain protein, FCHO2. *Genes Cells* **16**, 868-878, doi:10.1111/j.1365-2443.2011.01536.x (2011).

7 Ford, M. G. *et al.* Curvature of clathrin-coated pits driven by epsin. *Nature* **419**, 361-366, doi:10.1038/nature01020 (2002).

8 Boucrot, E. *et al.* Membrane fission is promoted by insertion of amphipathic helices and is restricted by crescent BAR domains. *Cell* **149**, 124-136, doi:10.1016/j.cell.2012.01.047 (2012).

9 Henne, W. M. *et al.* FCHo proteins are nucleators of clathrin-mediated endocytosis. *Science* **328**, 1281-1284, doi:10.1126/science.1188462 (2010).

10 Almeida, P. F., Vaz, W. L. & Thompson, T. E. Lipid diffusion, free area, and molecular dynamics simulations. *Biophys J* **88**, 4434-4438, doi:10.1529/biophysj.105.059766 (2005).

11 Pylypenko, O., Lundmark, R., Rasmuson, E., Carlsson, S. R. & Rak, A. The PX-BAR membrane-remodeling unit of sorting nexin 9. *EMBO J* **26**, 4788-4800, doi:10.1038/sj.emboj.7601889 (2007).

12 Henne, W. M. *et al.* Structure and analysis of FCHo2 F-BAR domain: a dimerizing and membrane recruitment module that effects membrane curvature. *Structure* **15**, 839-852, doi:10.1016/j.str.2007.05.002 (2007).

13 Hyman, J., Chen, H., Di Fiore, P. P., De Camilli, P. & Brunger, A. T. Epsin 1 undergoes nucleocytosolic shuttling and its eps15 interactor NH(2)-terminal homology (ENTH) domain, structurally similar to Armadillo and HEAT repeats, interacts with the transcription factor promyelocytic leukemia Zn(2)+ finger protein (PLZF). *J Cell Biol* **149**, 537-546 (2000).
